# Protective effects of berbamine against arginase-1 deficiency-induced injury in human brain microvascular endothelial cells

**DOI:** 10.3389/fphar.2024.1497973

**Published:** 2025-01-09

**Authors:** Xiaolan Wei, Weiwei Li, Zixuan Chen, Jintu Chen, Yun Chen, Jiangping Cai, Huasong Lin

**Affiliations:** ^1^ Department of Neurology, Quanzhou First Hospital Affiliated to Fujian Medical University, Quanzhou, Fujian, China; ^2^ Department of Cardiology, The Second Affiliated Hospital of Fujian Medical University, Quanzhou, Fujian, China; ^3^ Department of Clinical Laboratory, Quanzhou First Hospital Affiliated to Fujian Medical University, Quanzhou, Fujian, China; ^4^ Department of Geriatric Medicine, The Seventh Affiliated Hospital, Sun Yat-sen University, Shenzhen, Guangdong, China

**Keywords:** cerebral small vessel disease, arginase 1, arginine, berbamine, human brain microvascular endothelial cells

## Abstract

Endothelial cell dysfunction plays a crucial role in the early development of cerebral small vessel disease (CSVD). Arginase-1 (ARG1) is expressed in endothelial cells, and its deficiency may exacerbate cerebrovascular damage by increasing reactive oxygen species (ROS) production, thereby inducing endothelial cell apoptosis. Berbamine (BBM) has shown potential in neuroprotection and cardiovascular disease prevention. This study aimed to investigate the impact of ARG1 deficiency on human brain microvascular endothelial cells and the protective effects of BBM against ARG1 deficiency-induced damage. Human brain microvascular endothelial cells (HCMEC/D3) were cultured *in vitro*, and ARG1 knockdown or overexpression was achieved using plasmid transfection techniques. We examined the effects of ARG1 expression levels on HCMEC/D3 cell viability, migration, apoptosis, adhesion, and angiogenesis through cellular experiments. Additionally, we explored how ARG1 expression levels influenced arginine (Arg), nitric oxide (NO), and ROS levels in HCMEC/D3 cells. The results demonstrated that ARG1 deficiency inhibited HCMEC/D3 cell viability, migration, adhesion, and angiogenesis, while promoting apoptosis and elevating Arg, NO, and ROS levels in HCMEC/D3 cells. Next, the effect of different BBM concentrations on HCMEC/D3 cell viability was assessed using the CCK-8 assay, revealing that BBM at a concentration of 5 µM had no significant impact on cell viability. Subsequently, after successfully knocking down ARG1 in HCMEC/D3 cells, the cells were treated with BBM. The results showed that BBM effectively mitigated the negative effects of ARG1 deficiency on HCMEC/D3 cell viability, migration, apoptosis, adhesion, and angiogenesis, while also reducing Arg, NO, inducible nitric oxide synthase (iNOS), and ROS levels in HCMEC/D3 cells. In conclusion, this study suggests that ARG1 deficiency may damage HCMEC/D3 cells by increasing Arg levels, leading to elevated NO and ROS levels. BBM may provide protection to ARG1-deficient HCMEC/D3 cells by reducing Arg, NO, iNOS, and ROS levels. These findings deepen our understanding of the pathogenesis of CSVD and provide a theoretical basis for the clinical application of BBM.

## 1 Introduction

Cerebral small vessel disease (CSVD) is a clinical, imaging, and pathological syndrome resulting from various etiologies that affect small arteries (diameter <400 µm), their distal branches, arterioles, capillaries, and venules within the brain (Li et al., 2024). The main features of CSVD include white matter hyperintensities, lacunar infarcts, cerebral microbleeds, and enlarged perivascular spaces (Hainsworth et al., 2017). The incidence of CSVD increases exponentially with age, making it a major risk factor for acute stroke and cognitive decline (Cifu et al., 2023). However, the pathogenesis of CSVD remains incompletely understood, and no specific preventive or therapeutic strategies have been established. Research indicates that hypoperfusion, endothelial dysfunction, and blood-brain barrier (BBB) disruption are key pathological mechanisms in CSVD (Shi et al., 2024), closely associated with various vascular risk factors, particularly aging, hypertension, and smoking (Chen et al., 2024). Among these, BBB disruption occurs early in CSVD and is strongly correlated with stroke, cognitive impairment, white matter lesions, and lacunar infarcts in CSVD patients (Nation et al., 2019; Wardlaw et al., 2019). Thus, BBB dysfunction is considered a crucial pathological hallmark of CSVD (Abdul Hamid et al., 2024). The BBB is primarily composed of human brain microvascular endothelial cells (HBMECs) and their tight junctions, which work in concert with astrocytes, pericytes, and perivascular microglia to maintain BBB integrity (Yang et al., 2021). When endothelial cells are damaged, the integrity of the BBB is compromised, suggesting that preserving endothelial cell function could help maintain BBB integrity (Li et al., 2021). Therefore, protecting endothelial cell function may represent a potential therapeutic strategy for CSVD.

Arginase-1 (ARG1) was first discovered *in vivo* in the early 20th century (Zhang et al., 2020). It is a manganese metalloproteinase that plays a critical role in the hepatic urea cycle, converting arginine into urea and L-ornithine (Ming et al., 2020). L-ornithine and its metabolites, such as putrescine, spermidine, and spermine, are crucial for cell growth, proliferation, wound healing, tissue repair, and neurodevelopment. Therefore, ARG1 is integral to cellular functions and various metabolic pathways (Li et al., 2001; You et al., 2018). Previous studies have demonstrated that the classic function of ARG1 is its ability to compete with nitric oxide synthase (NOS) for the substrate arginine, thereby inhibiting nitric oxide (NO) production (Caldwell et al., 2015; Zhang et al., 2020). As an active molecule, NO interacts with reactive oxygen species (ROS), promoting oxidative stress (Barros et al., 2024). Consequently, ARG1 may reduce intracellular ROS levels by inhibiting NO production, thereby alleviating oxidative stress (Cai et al., 2019). In CSVD, endothelial cells, which contain numerous mitochondria, produce high levels of ROS. These ROS can disrupt the blood-brain barrier, impair endothelial function, and contribute to vascular degeneration, which may be part of the pathogenesis of CSVD (Lloret et al., 2021; Zhang et al., 2024). Additionally, ARG1 is expressed in endothelial cells (Raffort et al., 2019), and its deficiency leads to disturbances in arginine metabolism, affecting NO production and causing vascular dysfunction and inflammation (Gogiraju et al., 2022). ARG1 deficiency may also exacerbate cerebrovascular damage by increasing ROS production and inducing endothelial cell apoptosis (Morris et al., 2007). These findings suggest that ARG1 deficiency may impair HBMECs and thereby promote the progression of CSVD.

In recent years, natural plant compounds have garnered significant attention for their potential in the prevention and treatment of cerebrovascular diseases. Berbamine (BBM), a natural active bisbenzylisoquinoline alkaloid extracted from Berberidaceae plants, exhibits notable pharmacological activities (Peng et al., 2024; Tang et al., 2024), such as promoting hematopoiesis and increasing blood cell counts (Xu et al., 2022). Clinically, BBM is widely used to treat leukopenia induced by chemotherapy and/or radiotherapy, with minimal side effects (Liu et al., 2021). Furthermore, studies have shown that BBM possesses a range of pharmacological activities including antioxidant, anti-inflammatory, anti-tumor, immunomodulatory, and cardioprotective effects (Yin et al., 2022). As a recognized antioxidant, BBM demonstrates significant antioxidant properties in cardiovascular, metabolic, and neurodegenerative diseases (Xu et al., 2022), and holds potential for neuroprotection and cardiovascular disease prevention (Saranya et al., 2019). However, research on the effects of BBM on injured HBMECs remains limited.

Based on this, the aim of the present study is to investigate the impact of ARG1 deficiency on HBMECs and to further evaluate the protective effects of BBM on ARG1-deficient HBMECs. This research will provide new insights into the pathogenesis of CSVD and offer theoretical support for the clinical application of BBM, while also exploring novel approaches for the treatment of CSVD.

## 2 Materials and methods

### 2.1 Cell culture

The human brain microvascular endothelial cell line HCMEC/D3 was purchased from iCell Bioscience Inc. (Shanghai, China). HBMECs were cultured in endothelial cell-specific medium (iCell Bioscience Inc., Shanghai, China) containing 10% fetal bovine serum and 1% penicillin-streptomycin, and incubated at 37°C with 5% CO_2_ and saturated humidity.

### 2.2 Cell grouping and treatment

In experiments investigating the effects of ARG1 expression levels on HCMEC/D3 cells, the cells were randomly divided into the following groups: Control (no treatment), siNC (transfected with siRNA scramble), siARG1 (transfected with siARG1), NC-OE [transfected with the empty vector pcDNA3.1 (+)], and ARG1-OE [transfected with pCDNA3.1 (+)-ARG1]. The treatment methods for the siNC, siARG1, NC-OE, and ARG1-OE groups are detailed in Section 2.3. After transfection, cells from each group were collected for subsequent experiments.

In experiments investigating the effects of BBM on HCMEC/D3 cell viability, the cells were randomly divided into the following groups: Control, BBM (0.625 μM), BBM (1.25 μM), BBM (2.5 μM), BBM (5 μM), BBM (10 μM), BBM (20 μM), BBM (40 μM), BBM (80 μM), and BBM (100 μM). Each group of cells was plated in a 96-well plate containing media with different concentrations of BBM (0, 0.625, 1.25, 2.5, 5, 10, 20, 40, 80, and 100 μM) (MedChemExpress, NJ, United States). After 24 h of incubation, the cells were collected for subsequent experimental analysis.

In experiments investigating the effects of BBM in ARG1-deficient HCMEC/D3 cells, the cells were randomly divided into the following groups: siNC, siARG1, and siARG1 + BBM. The transfection methods for each group followed those described in Section 2.3. In the siARG1 + BBM group, after transfection, cells were transferred to medium containing 5 μM BBM and cultured for an additional 48 h.

### 2.3 Cell transfection

To knock down ARG1 or achieve its overexpression, we designed and synthesized three siRNA sequences targeting human ARG1 (shown in Table 1) and inserted the human ARG1 coding sequence into the pCDNA3.1 (+) vector for overexpression. The primers used for plasmid construction were as follows: Forward, 5′-AAG​CTT​GCC​ACC​ATG​AGC​GCC​AAG​TCC​AGA-3'; Reverse, 5′-CTT​AAG​AAT​GAA​TCC​ACC​CAA​TTC-3'. Following the manufacturer’s instructions, jetPRIME^®^ Transfection Reagent (Polyplus) was used to transfect ARG1 siRNA or pCDNA3.1 (+)-ARG1 into HBMECs. Cells transfected with siRNA scramble control (siNC) and the empty pcDNA3.1 (+) vector (NC-OE) served as negative controls. After 48 h of transfection, ARG1 mRNA and protein expression levels in each group were analyzed using real-time polymerase chain reaction and Western blot assay.

**TABLE 1:** The sequences of siRNA.

Gene	Forward (5′-3′)	Reverse (5′-3′)
siARG1-1	5′-GGU​CUG​CUU​GAG​AAA​CUU​AAA​TT-3′	5′-UUU​AAG​UUU​CUC​AAG​CAG​ACC​TT-3′
siARG1-2	5′-GAC​UGA​AGU​GGA​CAG​ACU​AGG​TT-3′	5′-CCU​AGU​CUG​UCC​ACU​UCA​GUC​TT-3′
siARG1-2	5′-CCA​GAA​GAA​GUA​ACU​CGA​ACA​TT-3′	5′-UGU​UCG​AGU​UAC​UUC​UUC​UGG​TT-3′
siNC	5′-UUC​UCC​GAA​CGU​GUC​ACG​UTT-3′	5′-ACG​UGA​CAC​GUU​CGG​AGA​ATT-3′

### 2.4 Real-time polymerase chain reaction (RT-PCR)

The HCMEC/D3 cells from the Control, siNC, siARG1, NC-OE, ARG1-OE, and siARG1 + BBM groups, processed as described in Section 2.3, were seeded into 6-well plates (1×10^7^ cells per well), with at least three replicates per group. Total RNA was extracted using TRIzol reagent (TSINGKE, Beijing, China) and reverse transcription was performed using a reverse transcription kit. Real-time quantitative PCR was conducted using a real-time fluorescence quantitative PCR kit (TSINGKE, Beijing, China) according to the manufacturer’s instructions. GAPDH was used as the internal reference gene. The relative expression levels of ARG1 and iNOS in the cells were calculated using the 2^−ΔΔCT^ method (Gong et al., 2024). Primer sequences are listed in Table 2.

**TABLE 2:** The primer sequence.

Gene	Forward (5′-3′)	Reverse (5′-3′)
ARG1	5′-TCA​AGA​AGA​ACG​GAA​GAA​TCA​GC-3′	5′-TCA​GGA​GGA​AAG​ATA​CAG​GTT​GT-3′
iNOS	5′-CAG​CAC​ATT​CAG​ATC​CCC​AAG-3′	5′-CGG​ACT​TTG​TAG​ATT​CTG​CCG-3′
GAPDH	5′-TCA​AGA​AGG​TGG​TGA​AGC​AGG-3′	5′-TCA​AAG​GTG​GAG​GAG​TGG​GT-3′

### 2.5 Western blot assay

HCMEC/D3 cells from the Control, siNC, siARG1, NC-OE, ARG1-OE, and siARG1 + BBM groups, processed as described in Section 2.3, were cultured in 60 mm culture dishes (1 × 10^7^ cells per dish). After protein extraction from the HCMEC/D3 cells using RIPA lysis buffer, the protein concentration was measured using a BCA protein assay kit (Beyotime, Shanghai, China). Approximately 20 µg of the cell lysates were separated by electrophoresis and transferred to a polyvinylidene fluoride (PVDF) membrane. After blocking with 5% non-fat milk, the membrane was incubated overnight at 4°C with diluted primary antibodies: anti-ARG1 (1:1,000, Proteintech, China, 66129-1-Ig), anti-iNOS (1:500, Proteintech, China, 18985-1-AP), and anti-GAPDH (1:5,000, Proteintech, China, 60004-1-Ig). The membrane was then incubated with the secondary antibody (1:10,000, Jackson, 111-035-003) at room temperature for 1 h. Enhanced Luminol Reagent and Oxidizing Reagent were mixed with ddH₂O and added to the membrane. The PVDF membrane was exposed to ECL substrate, and the results were observed using a gel imaging system. The protein bands were analyzed for their gray intensity, and the protein expression levels of ARG1 and iNOS were normalized to GAPDH (Thaweewattanodom et al., 2024).

### 2.6 Flow cytometry

Cell apoptosis was assessed using flow cytometry. First, cells from the Control, NC-OE, ARG1-OE, siNC, siARG1, and siARG1 + BBM groups were collected, and the supernatant was removed by centrifugation. The cells were then resuspended in 100 µL of 1 × Binding Buffer, followed by the addition of 5 µL of Annexin V-Alexa Fluor 647 and 10 µL of PI. The mixture was incubated in the dark at room temperature for 15 min. After incubation, 300 µL of 1 × Binding Buffer was added, and the samples were mixed and kept on ice. Within 1 h, the samples were analyzed using a flow cytometer (Beckman Coulter, United States).

### 2.7 Cell counting kit-8 assay

Cell viability was assessed using the Cell Counting Kit-8 (CCK-8) assay kit (Dojindo Molecular Technologies, Inc., Kumamoto, Japan). Briefly, HCMEC/D3 cells from the Control, NC-OE, ARG1-OE, siNC, siARG1, and BBM (0.625–100 μM) groups were cultured in 96-well plates. Following this, 10 µL of CCK-8 reagent was added to each well and the cells were incubated for 1 h. The optical density (OD) at 450 nm was then measured using a microplate reader.

### 2.8 Angiogenesis assay

The angiogenic ability of the cells was evaluated using an angiogenesis assay. Matrigel (10 µL per well) was added to the bottom of the culture plate, and after solidification, 50 μL of HCMEC/D3 cells in the logarithmic growth phase (2.5 × 10^5^ cells/mL) from the Control, NC-OE, ARG1-OE, siNC, siARG1, and siARG1 + BBM groups were added. The cell treatments were carried out as described in Section 2.3. After 4 h of incubation, images were taken, and the number of vascular branches was analyzed and compared using ImageJ.

### 2.9 Scratch wound-healing assay

Cells in the logarithmic growth phase from the Control, NC-OE, ARG1-OE, siNC, siARG1, and siARG1 + BBM groups were digested with trypsin and prepared into single-cell suspensions. The suspension was plated at a density of 5 × 10^4^ cells/well in a 96-well plate. After 24 h of incubation, a vertical scratch was made in each well using a 10 µL pipette tip. The cell culture medium was then aspirated, and the wells were washed three times with PBS to remove cell debris from the scratches. Serum-free medium was added to the wells. Experimental groups were assigned and corresponding treatments were applied according to the experimental design. Images of the treated cells were captured at 0, 24, and 48 h for statistical analysis.

### 2.10 Cell adhesion experiment

The processed cells from the Control, NC-OE, ARG1-OE, siNC, siARG1, and siARG1 + BBM groups were seeded into fibronectin-coated 96-well plates at a density of 4 × 10^4^ cells per well and incubated at 37°C for 3 h. After removing the medium containing non-adherent cells, 100 μL of serum-free medium was added to each well. Subsequently, 10 μL of MTT solution was added to each well, and the cells were incubated at 37°C for 4 h. The supernatant was then discarded, and 150 μL of DMSO was added to each well to dissolve the formazan crystals. The absorbance was measured at 570 nm using a microplate reader.

### 2.11 Arginine (Arg) content measurement

According to the manufacturer’s instructions, the arginine content in cells from the Control, NC-OE, ARG1-OE, siNC, siARG1, and siARG1 + BBM groups was measured using an arginine assay kit (Solarbio, Beijing, China). The absorbance at 525 nm was determined using a spectrophotometer (KAIAO, Beijing, China).

### 2.12 Measurement of NO content

According to the manufacturer’s instructions, the NO production in cells from the Control, NC-OE, ARG1-OE, siNC, siARG1, and siARG1 + BBM groups was measured using the Total Nitric Oxide Assay Kit (Beyotime, China).

### 2.13 ROS detection

According to the instructions of the ROS assay kit (Solarbio, Beijing, China), the culture medium of cells from the Control, NC-OE, ARG1-OE, siNC, siARG1, and siARG1 + BBM groups was discarded. Then, 200 μL of 10 μmol/L 2′,7′-dichlorodihydrofluorescein diacetate (DCFH-DA) working solution was added to fully cover the cells, followed by incubation at 37°C in the dark for 5 min. The plate was gently inverted to ensure sufficient contact between the probe and the cells. The cells were washed three times with PBS to thoroughly remove excess DCFH-DA that had not entered the cells. Finally, the cells were resuspended in 500 μL of PBS, and the ROS levels in each group were analyzed using a flow cytometer (Beckman Coulter, United States).

### 2.14 TUNEL detection

After washing the cells from the Control, NC-OE, ARG1-OE, siNC, siARG1, and siARG1 + BBM groups with PBS, the cells were fixed with 4% paraformaldehyde for 30 min and permeabilized with 0.3% Triton X-100 for 5 min. The cells were then incubated with 0.3% hydrogen peroxide for 20 min to block endogenous peroxidase activity. Subsequently, the cells were incubated with TUNEL solution (50 μL) at 37°C for 1 h. The stained cells were observed using a fluorescence microscope.

### 2.15 Statistical analysis

All experiments were performed in triplicate. Data are expressed as mean ± standard deviation. Statistical analyses were conducted using GraphPad Prism 9 (GraphPad Inc., San Diego, CA, United States). One-way analysis of variance (ANOVA) followed by Tukey’s *post hoc* test was used for comparisons among multiple groups. A value of *p* < 0.05 was considered statistically significant.

## 3 Results

### 3.1 ARG1 deficiency inhibits HCMEC/D3 cell viability and promotes cell apoptosis

**FIGURE 1:**
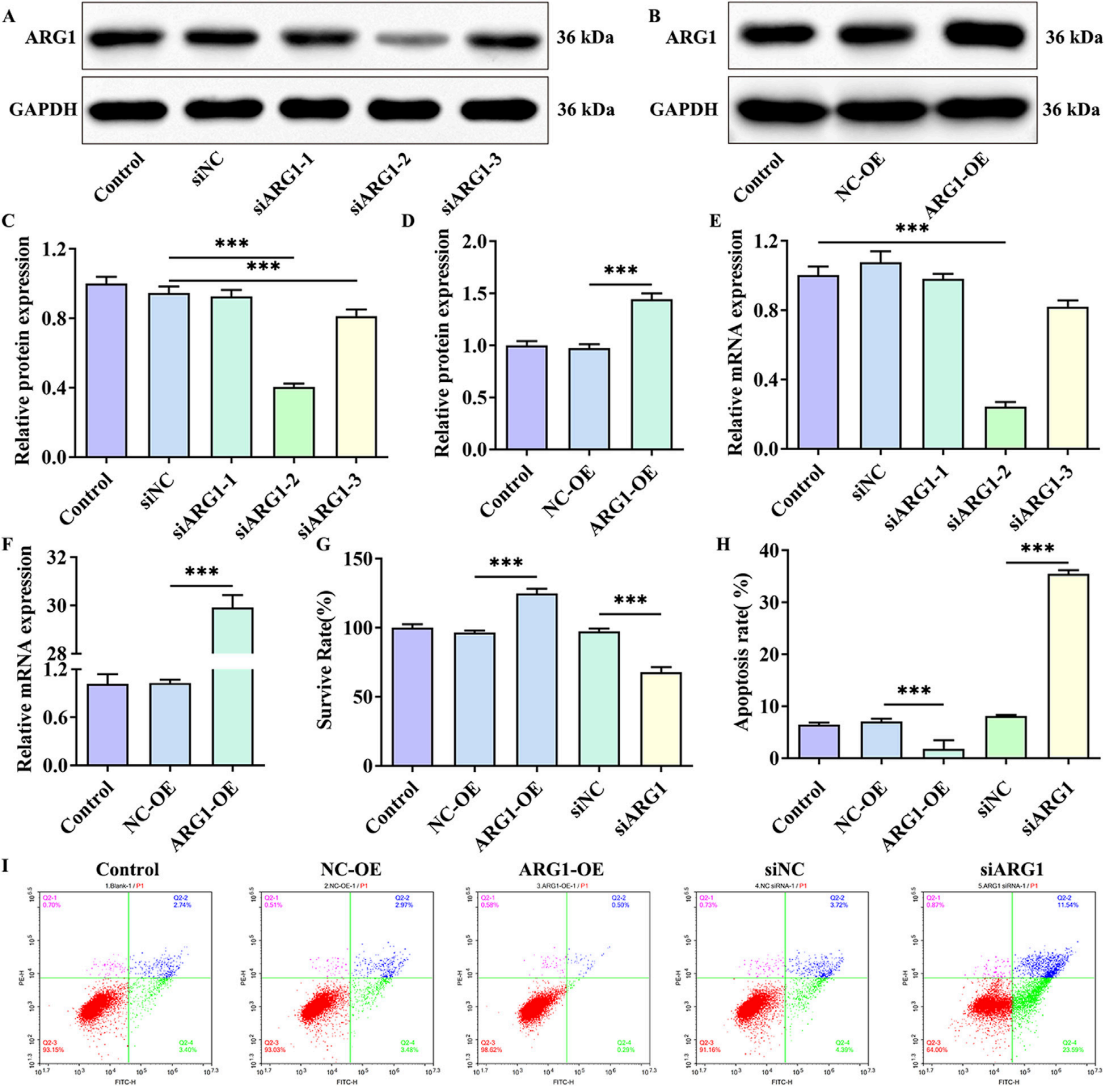
Effects of ARG1 deficiency on HCMEC/D3 cell viability and apoptosis. **(A)** Western blot assay assessing the efficiency of ARG1 knockdown. **(B)** Western blot assay evaluating the efficiency of ARG1 overexpression. **(C)** Statistical analysis of Western blot results for ARG1 knockdown efficiency. **(D)** Statistical analysis of Western blot results for ARG1 overexpression efficiency. **(E)** RT-PCR analysis of ARG1 knockdown efficiency. **(F)** RT-PCR analysis of ARG1 overexpression efficiency. **(G)** CCK-8 assay measuring cell viability. **(H, I)** Flow cytometry analysis of cell apoptosis. Control, Normal HCMEC/D3 cells. NC-OE, HCMEC/D3 cells transfected with the empty pcDNA3.1 (+) vector. ARG1-OE, HCMEC/D3 cells transfected with the pCDNA3.1 (+)-ARG1 vector. siNC, HCMEC/D3 cells transfected with siRNA scramble control. siARG1, HCMEC/D3 cells transfected with ARG1 siRNA. ^***^ *p* < 0.001.

Studies have shown that ARG1 is expressed in endothelial cells and plays crucial roles in various pathological processes, including angiogenesis, wound healing, and cancer (Raffort et al., 2019; Zakeri et al., 2024). To investigate the effects of ARG1 on HCMEC/D3 cells, we performed ARG1 knockdown and overexpression in these cells through plasmid transfection, and assessed transfection efficiency using RT-PCR and Western blot analysis. The RT-PCR and Western blot results (Figures 1A–F) demonstrated successful knockdown and overexpression of ARG1, with siARG1-2 showing the most effective knockdown, which was thus selected for subsequent experiments. Additionally, we evaluated the impact of ARG1 expression levels on HCMEC/D3 cell viability and apoptosis using CCK-8 assays and flow cytometry. The results indicated that ARG1 deficiency significantly inhibited HCMEC/D3 cell viability (Figure 1G, *p* < 0.001) and markedly promoted cell apoptosis (Figures 1H, I, *p* < 0.001), whereas ARG1 overexpression exhibited the opposite effects.

### 3.2 ARG1 deficiency inhibits angiogenesis, migration, and adhesion in HCMEC/D3 cells

**FIGURE 2:**
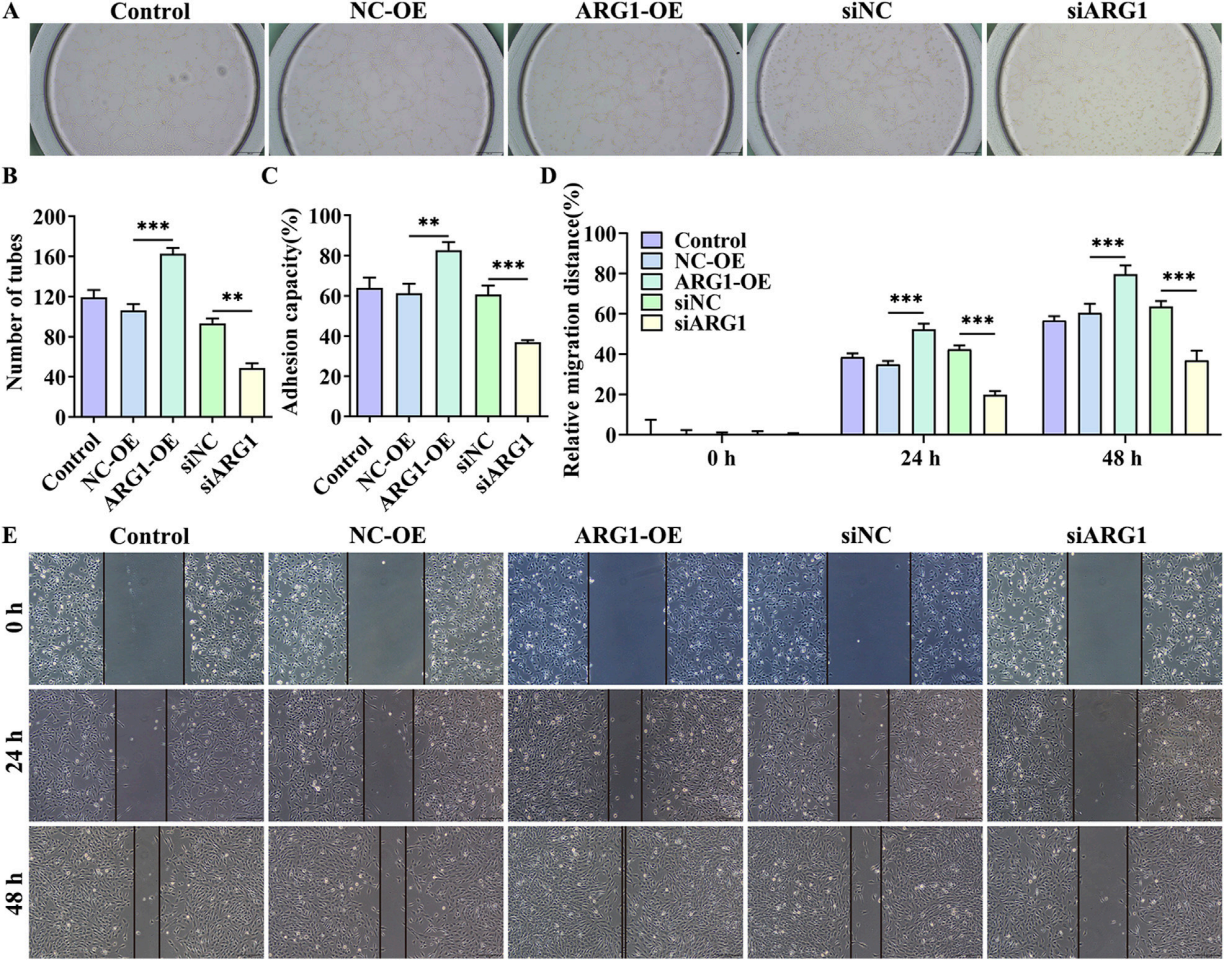
The effects of ARG1 deficiency on angiogenesis, migration, and adhesion in HCMEC/D3 cells. **(A, B)** Angiogenesis assay. **(C)** Cell adhesion assay. **(D, E)** Scratch assay. Control, Normal HCMEC/D3 cells. NC-OE, HCMEC/D3 cells transfected with the empty pcDNA3.1 (+) vector. ARG1-OE, HCMEC/D3 cells transfected with the pCDNA3.1 (+)-ARG1 vector. siNC, HCMEC/D3 cells transfected with siRNA scramble control. siARG1, HCMEC/D3 cells transfected with ARG1 siRNA. ^**^ *p* < 0.01, ^***^ *p* < 0.001.

Angiogenesis is a distinctive feature of endothelial cells (Li et al., 2024). To investigate the impact of ARG1 on endothelial cell angiogenesis, we conducted angiogenesis assays. The results show that (Figures 2A, B), compared to the siNC group, the siARG1 group exhibited reduced and incomplete tubular structure formation (*p* < 0.01). In contrast, compared to the NC-OE group, the ARG1-OE group demonstrated increased and complete tubular structures (*p* < 0.001), indicating that ARG1 promotes angiogenesis. Additionally, cell scratch and adhesion assay results (Figures 2C–E) revealed that ARG1 deficiency inhibited cell migration and adhesion, while ARG1 overexpression enhanced these processes. In summary, these results indicate that ARG1 deficiency suppresses angiogenesis, migration, and adhesion in HCMEC/D3 cells.

### 3.3 ARG1 deficiency increases arginine levels in HCMEC/D3 cells while upregulating NO and ROS levels, thereby promoting cell apoptosis

**FIGURE 3:**
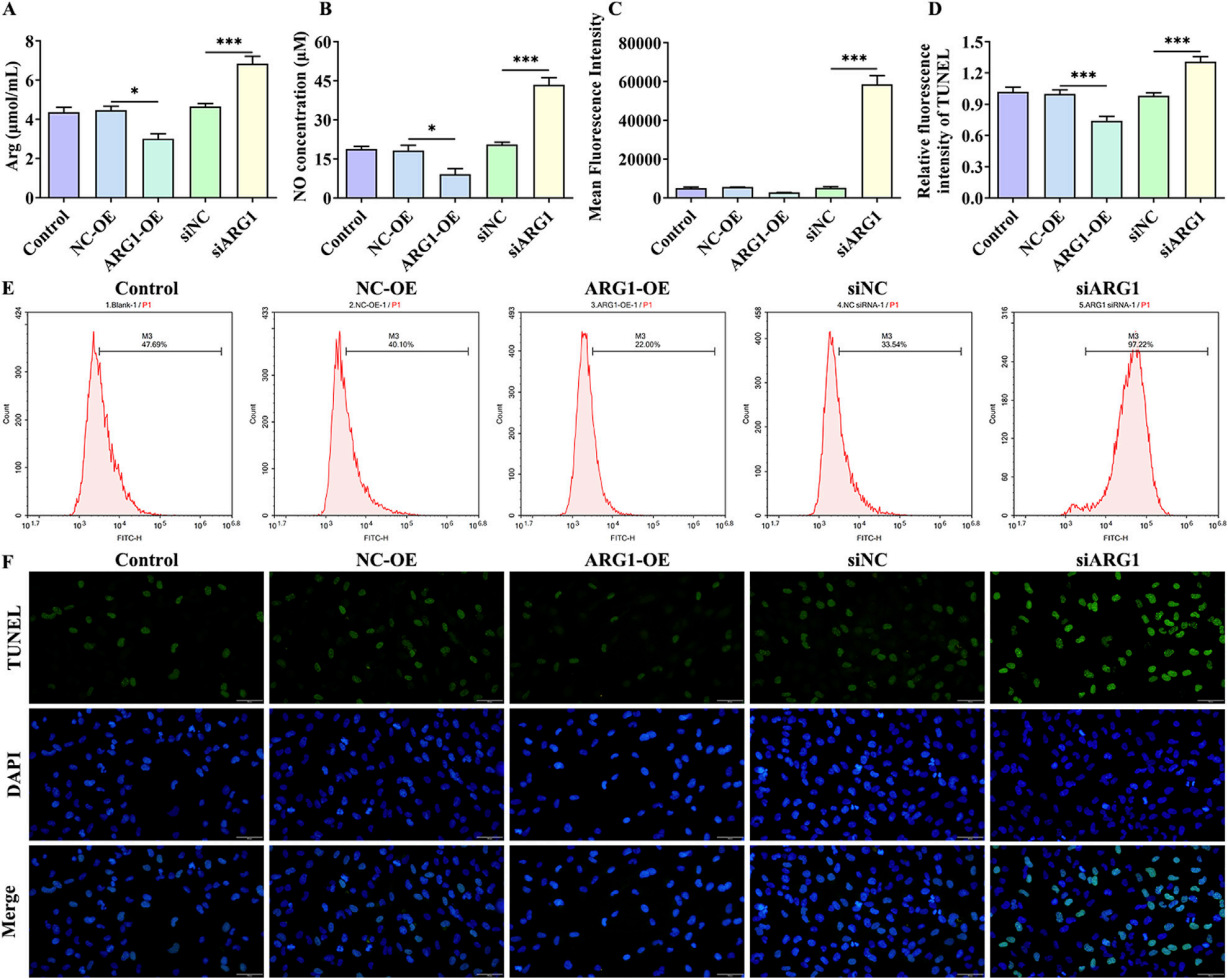
Impact of ARG1 deficiency on arginine, NO, and ROS levels, and apoptosis in HCMEC/D3 cells. **(A)** Measurement of arginine levels in cells. **(B)** Measurement of NO levels in cells. **(C)** Statistical analysis of ROS levels detected by flow cytometry. **(D)** TUNEL fluorescence level statistics. **(E)** Detection of ROS levels in cells using flow cytometry. **(F)** Detection of cell apoptosis using the TUNEL assay. Control, Normal HCMEC/D3 cells. NC-OE, HCMEC/D3 cells transfected with the empty pcDNA3.1 (+) vector. ARG1-OE, HCMEC/D3 cells transfected with the pCDNA3.1 (+)-ARG1 vector. siNC, HCMEC/D3 cells transfected with siRNA scramble control. siARG1, HCMEC/D3 cells transfected with ARG1 siRNA. ^*^ *p* < 0.05, ^***^ *p* < 0.001. Arg, arginine. NO, nitric oxide.

ARG1 inhibits NO production by competing with NOS for arginine, converting it into urea and ornithine (Zhao et al., 2024). Abnormal NO synthesis has been closely associated with CSVD and BBB damage (Liao et al., 2021). To investigate the impact of ARG1 expression levels on arginine and NO in HCMEC/D3 cells, we measured arginine and NO levels. The results (Figures 3A, B) indicate that ARG1 knockdown significantly increased the levels of arginine and NO in HCMEC/D3 cells (*p* < 0.001), whereas ARG1 overexpression significantly reduced these levels (*p* < 0.05). This suggests that ARG1 deficiency leads to elevated arginine and NO levels in HCMEC/D3 cells. Furthermore, elevated ROS levels are closely linked to the occurrence and progression of CSVD (Lloret et al., 2021). Under ROS conditions, superoxide rapidly combines with NO to form peroxynitrite (Meza et al., 2019), a reactive molecule that can damage proteins and DNA, inducing cell death (Xie et al., 2005). To further explore the effects of ARG1 deficiency on ROS levels and cell apoptosis in HCMEC/D3 cells, we conducted flow cytometry and TUNEL assays. The results (Figures 3C–F) showed that, compared to the siNC group, the ROS fluorescence levels and TUNEL fluorescence levels in the siARG1 group were significantly increased (*p* < 0.001). Compared to the NC-OE group, the ROS fluorescence levels and TUNEL fluorescence levels in the ARG1-OE group were reduced. These findings indicate that ARG1 knockdown not only significantly increases ROS levels in HCMEC/D3 cells but also promotes cell apoptosis, whereas ARG1 overexpression has the opposite effect. These results imply that ARG1 deficiency might promote apoptosis in HCMEC/D3 cells, potentially through increased arginine levels and the upregulation of NO and ROS.

### 3.4 BBM effectively alleviates the damage to HCMEC/D3 cells caused by ARG1 deficiency

**FIGURE 4:**
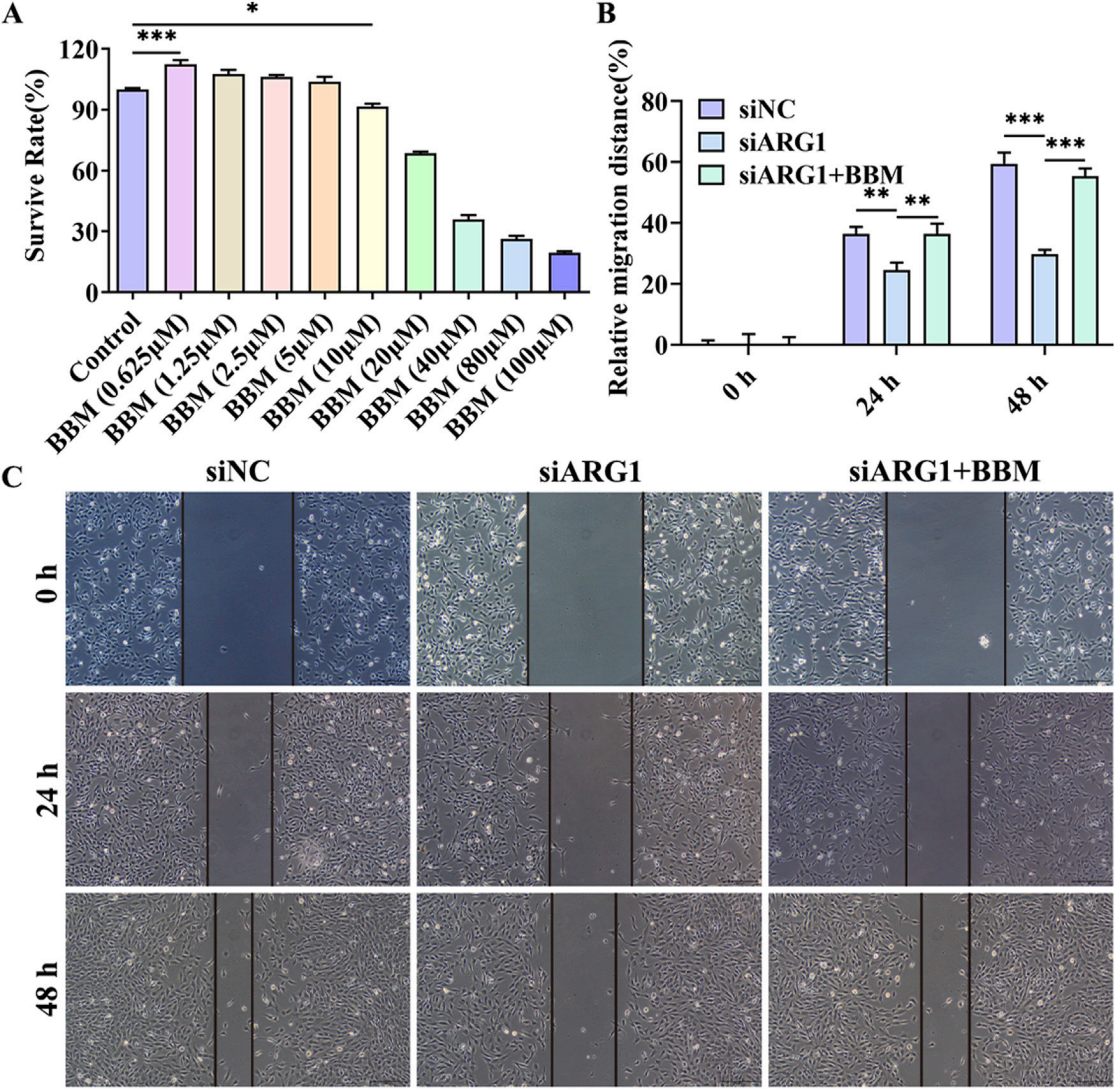
Effect of BBM on the viability and migration of ARG1-deficient HCMEC/D3 cells. **(A)** CCK-8 assay to evaluate the impact of BBM on HCMEC/D3 cell viability. **(B, C)** Scratch assay to assess cell migration. Control, Normal HCMEC/D3 cells. BBM (0.625–100 μM), HCMEC/D3 cells treated with BBM in culture media containing 0.625–100 μM BBM. siNC, HCMEC/D3 cells transfected with siRNA scramble control. siARG1, HCMEC/D3 cells transfected with ARG1 siRNA. siARG1 + BBM: HCMEC/D3 cells were transfected with siRNA scramble control and then treated with 5 μM BBM in culture media. ^*^ *p* < 0.05, ^**^ *p* < 0.01, ^***^ *p* < 0.001.

The chemical structure of BBM is shown in Supplementary Figure S1. To investigate the protective effects of BBM on HCMEC/D3 cell damage induced by ARG1 deficiency, we first assessed the impact of BBM on HCMEC/D3 cell viability using the CCK-8 assay to determine its safe dosage. The results (Figure 4A) indicated that BBM at a concentration of 5 μM had no significant effect on HCMEC/D3 cell viability and actually improved cell viability. However, when the concentration of BBM was increased to 10 μM, a significant reduction in HCMEC/D3 cell viability was observed (*p* < 0.05). Therefore, for subsequent experiments, we determined the concentration of BBM to be 5 μM.

**FIGURE 5:**
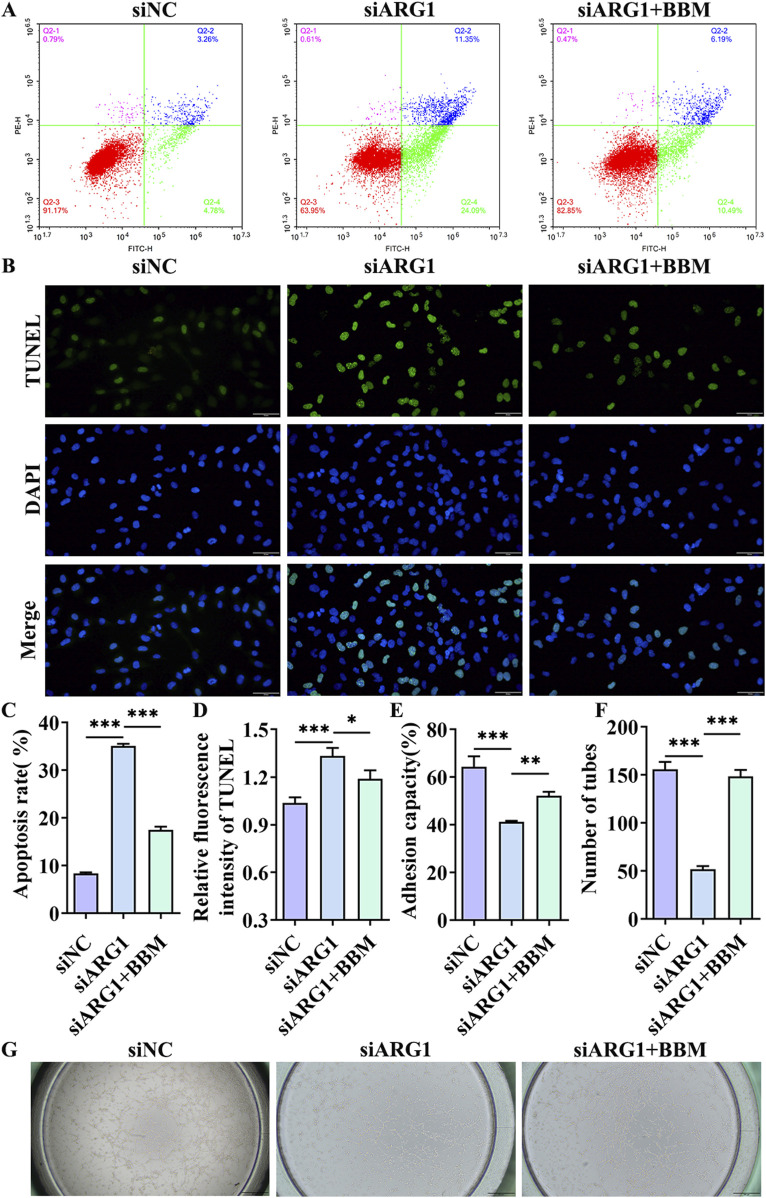
The effect of BBM on apoptosis, cell adhesion, and angiogenesis in HCMEC/D3 cells with ARG1 deficiency. **(A)** Apoptosis analysis by flow cytometry. **(B)** Apoptosis detection by TUNEL assay. **(C)** Statistical analysis of apoptosis by flow cytometry. **(D)** TUNEL fluorescence level statistics. **(E)** Cell adhesion assay. **(F, G)** Angiogenesis assay. siNC, HCMEC/D3 cells transfected with siRNA scramble control. siARG1, HCMEC/D3 cells transfected with ARG1 siRNA. siARG1 + BBM: HCMEC/D3 cells were transfected with siRNA scramble control and then treated with 5 μM BBM in culture media. ^*^ *p* < 0.05, ^**^ *p* < 0.01, ^***^ *p* < 0.001.

This study has demonstrated that ARG1 deficiency inhibits migration, angiogenesis, and adhesion of HCMEC/D3 cells while promoting apoptosis. To investigate the role of BBM in HCMEC/D3 cells with ARG1 knockdown, we transfected these cells with ARG1-targeting plasmids and concurrently treated them with BBM. The results of the scratch assay (Figures 4B, C) revealed a significant enhancement in cell migration in the siARG1 + BBM group compared to the siARG1 group (*p* < 0.01). Flow cytometry and TUNEL assays (Figures 5A–D) indicated a reduction in apoptosis in the siARG1 + BBM group compared to the siARG1 group. Furthermore, both the angiogenesis and cell adhesion assays (Figures 5E–G) showed improved angiogenic and adhesive capabilities in the siARG1 + BBM group relative to the siARG1 group. These findings suggest that BBM can effectively reverse the impairments in migration, angiogenesis, and adhesion induced by ARG1 deficiency and significantly inhibit ARG1 deficiency-induced apoptosis.

**FIGURE 6:**
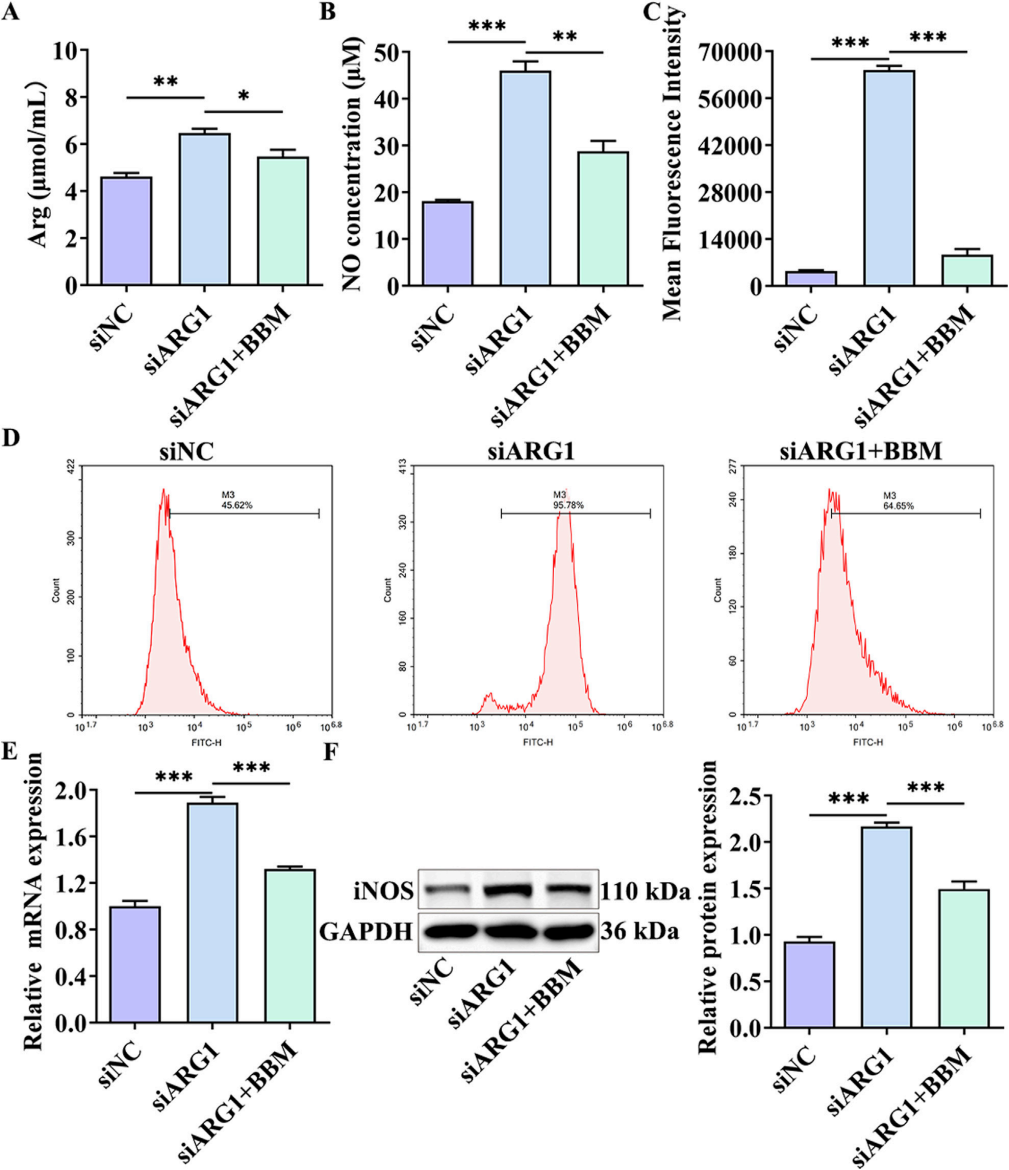
The effects of BBM on Arg, NO, ROS, and iNOS levels in ARG1-deficient HCMEC/D3 cells. **(A)** Arg content analysis. **(B)** NO content analysis. **(C, D)** Flow cytometry analysis of intracellular ROS levels. **(E)** RT-PCR analysis of iNOS levels. **(F)** Western blot analysis of iNOS levels. siNC, HCMEC/D3 cells transfected with siRNA scramble control. siARG1, HCMEC/D3 cells transfected with ARG1 siRNA. siARG1 + BBM: HCMEC/D3 cells were transfected with siRNA scramble control and then treated with 5 μM BBM in culture media. ^*^ *p* < 0.05, ^**^ *p* < 0.01, ^***^ *p* < 0.001. Arg, arginine. NO, nitric oxide.

BBM is a bioactive compound with antioxidant properties, known to reduce levels of ROS and the expression of iNOS, an essential enzyme in NO production (Sithuraj et al., 2018). Additionally, Arg is a key substrate for NO synthesis (Yu et al., 2023). To investigate the effects of BBM on Arg, NO, ROS, and iNOS in ARG1-deficient HCMEC/D3 cells, we performed assays to measure Arg and NO levels, flow cytometry, RT-PCR, and Western blot analysis. The results (Figure 6) indicated that, compared to the siARG1 group, the siARG1 + BBM group exhibited decreased levels of Arg, NO, ROS, and iNOS. These findings suggest that BBM can inhibit the accumulation of Arg caused by ARG1 deficiency and downregulate NO, ROS, and iNOS levels within the cells.

## 4 Discussion

**FIGURE 7:**
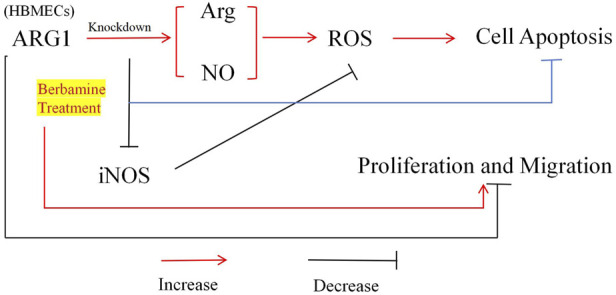
Mechanism of BBM’s protective effect on human cerebral microvascular endothelial cell injury.

Early pathological changes in CSVD are primarily characterized by endothelial dysfunction, and drugs that stabilize endothelial cell function hold promise for mitigating white matter vulnerability associated with CSVD damage (Rajani et al., 2018). Although the loss of ARG1 has been shown to induce endothelial cell apoptosis and exacerbate cerebrovascular damage, its specific role in microvascular endothelial cell injury remains unclear (Morris et al., 2007). BBM is a compound with various pharmacological activities, including anti-inflammatory, antioxidant, antitumor, and antibacterial effects (Liu et al., 2021), and shows potential for neuroprotection as well as the prevention and treatment of cardiovascular diseases (Saranya et al., 2019). Therefore, to investigate the role of ARG1 deficiency in brain microvascular endothelial cell injury and the protective effects of BBM, we first modulated ARG1 expression in HCMEC/D3 through plasmid transfection to either knockdown or overexpress the gene. Our experimental results (Figure 7) demonstrate that ARG1 deficiency significantly inhibits cell viability, migration, adhesion, and angiogenesis of HCMEC/D3 cells, while promoting apoptosis and increasing levels of Arg, ROS, and NO in these cells. Further experiments revealed that BBM effectively alleviates the damage caused by ARG1 deficiency in HCMEC/D3 cells. These findings suggest that ARG1 may serve as a potential therapeutic target for CSVD, while BBM could be a promising therapeutic strategy for managing this condition.

Arg is an essential amino acid that serves as a critical substrate for protein synthesis and as a precursor for various molecules involved in cell signaling and metabolic functions, such as NO, proline, and polyamines (Yu et al., 2023). Arginase (ARG) and NOS are key enzymes involved in Arg metabolism (Hu et al., 2024). ARG converts Arg into L-ornithine and urea, whereas NOS synthesizes NO and L-citrulline from Arg (Li et al., 2001). Interestingly, ARG and NOS share Arg as a common substrate, and inhibiting ARG activity could potentially increase Arg availability, thereby promoting NO production (Tratsiakovich et al., 2013). Consistent with this, our study found that ARG1 deficiency led to increased levels of Arg and NO in HCMEC/D3 cells. This ARG1 deficiency was also associated with elevated levels of ROS, which may be related to the increased NO levels. As a reactive nitrogen species (RNS), NO is produced by damaged mitochondria via iNOS and can alter mitochondrial respiratory function, biosynthesis, and oxidative stress by increasing ROS/RNS production (Upadhyay and Batuman, 2022). Moreover, as a reactive molecule, NO can interact with other ROS, thereby promoting oxidative stress or participating in redox signaling (Barros et al., 2024). Studies have shown that under oxidative stress conditions, superoxide rapidly combines with NO to form peroxynitrite, leading to NO depletion and ultimately resulting in endothelial dysfunction (Meza et al., 2019). Peroxynitrite has been reported to be a potent oxidant capable of damaging proteins and DNA, potentially exacerbating vascular dysfunction (Xie et al., 2005). Therefore, we speculate that in HCMEC/D3 cells, ARG1 deficiency may elevate Arg levels, subsequently increasing NO production, leading to elevated ROS levels, thereby inhibiting cell viability, migration, and angiogenesis, and promoting apoptosis.

Another important finding of this study is that BBM exhibits a protective effect against the damage induced by ARG1 deficiency in HCMEC/D3 cells. BBM is a natural bioactive molecule with significant effects in cardiovascular, metabolic, and neurodegenerative diseases due to its antioxidant properties (Xu et al., 2022). However, no studies have been conducted on the role of BBM in CSVD thus far. Therefore, based on BBM’s antioxidant characteristics, we investigated its effects on ROS, NO, Arg, and iNOS levels in ARG1-deficient HCMEC/D3 cells, as well as its regulatory role in cell viability, migration, adhesion, apoptosis, and angiogenesis. The results showed that BBM alleviated the negative effects of ARG1 deficiency in HCMEC/D3 cells and significantly reduced the levels of Arg, NO, ROS, and iNOS. Evidence suggests that BBM exhibits effective O^2−^ scavenging properties (Muhammad et al., 2024), which can modulate ROS generation in various diseases, including diabetes, cancer, and inflammation (Kumar et al., 2015). A study by Sharma A et al. showed that under oxidative stress conditions, Nrf2 translocates to the nucleus, activating the ARE-dependent antioxidant mechanisms. BBM treatment increased the nuclear accumulation of Nrf2 and upregulated the expression levels of HO-1, Nqo-1, SOD2, and catalase, thereby reducing ROS production (Sharma et al., 2020). A similar phenomenon was observed in our study, where BBM significantly reduced ROS levels in ARG1-deficient HCMEC/D3 cells, suggesting that the modulation of ROS levels by BBM in these cells may be related to the Nrf2 antioxidant pathway (Zhang et al., 2023). Additionally, a study by Faheem M et al. found that in diabetic neuropathy, BBM could downregulate NO and iNOS levels and inhibit NF-κB expression (Faheem et al., 2022). NF-κB regulates the production of NO by binding to specific sites on the iNOS promoter region (Tiwari et al., 2024). In this study, we found that BBM effectively suppressed NO and iNOS levels in ARG1-deficient HCMEC/D3 cells, suggesting that BBM may reduce NO generation by negatively regulating the NF-κB signaling pathway, thereby decreasing ROS levels. However, further studies are needed to validate these speculations. In conclusion, these results suggest that BBM may exert a protective effect on ARG1-deficient HCMEC/D3 cells by modulating the levels of Arg, NO, and ROS.

This study also has several limitations. First, the protective effect of BBM against the damage caused by ARG1 deficiency in HCMEC/D3 cells was only validated at the cellular level, without further verification through animal experiments or clinical studies to strengthen the current findings. Second, the protective mechanism of BBM in HCMEC/D3 cells with ARG1 deficiency may involve other, yet unidentified, pathways. In future research, we aim to explore these potential mechanisms in greater depth to provide more robust evidence supporting the clinical application of BBM.

## 5 Conclusion

To the best of our knowledge, this study is the first to investigate the role of ARG1 in CSVD and the effects of BBM on ARG1-deficient HCMEC/D3 cells. Our findings indicate that ARG1 deficiency induces damage to HCMEC/D3 cells, likely by regulating Arg expression and subsequently affecting the levels of NO and ROS. This results in reduced cell viability, migration, adhesion, and angiogenesis, as well as increased apoptosis. Furthermore, BBM shows a significant protective effect against HCMEC/D3 cell damage induced by ARG1 deficiency by modulating the levels of Arg, NO, and ROS. These discoveries not only deepen our understanding of the pathogenesis of CSVD but also provide a theoretical basis for the clinical application of BBM. This study offers a new perspective for further exploring the specific molecular mechanisms of CSVD and lays the foundation for developing innovative BBM-based therapeutic strategies. Such strategies hold promise for providing more effective treatment options for CSVD patients, thereby significantly improving their quality of life.

## Data Availability

The original contributions presented in the study are included in the article/Supplementary Material, further inquiries can be directed to the corresponding authors.
