# Supplementary figures and images for: Protective effects of berbamine against arginase-1 deficiency-induced injury in human brain microvascular endothelial cells
Source: Front Pharmacol. 2025 Jan 9;15:1497973. doi: 10.3389/fphar.2024.1497973 (PMC11754418; doi:10.3389/fphar.2024.1497973)

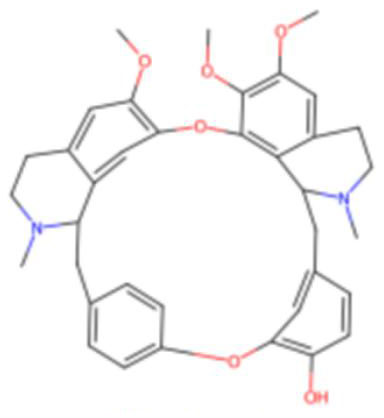


**Supplementary Figure S1.** The chemical structural formula of berbamine.

Supplement: Supplementary file 1 [file DataSheet1.docx]
